# Collagen induced arthritis increases secondary metastasis in MMTV-PyV MT mouse model of mammary cancer

**DOI:** 10.1186/1471-2407-11-365

**Published:** 2011-08-22

**Authors:** Lopamudra Das Roy, Sriparna Ghosh, Latha B Pathangey, Teresa L Tinder, Helen E Gruber, Pinku Mukherjee

**Affiliations:** 1 University of North Carolina at Charlotte, Dept. of Biology, 9201 University City Blvd., NC 29223,USA; 2 Carolinas Medical Center, Light Microscopy and Imaging Center, NC 28232, USA; 3 Mayo Clinic in Arizona, Department of Immunology, 13400 E. Shea Blvd. Scottsdale, AZ 85259, USA; 4 Carolinas Medical Center, Department of Orthopedic Surgery, NC 28232, USA

## Abstract

**Background:**

Several studies have demonstrated that sites of chronic inflammation are often associated with the establishment and growth of various malignancies. A common inflammatory condition in humans is autoimmune arthritis (AA). Although AA and cancer are different diseases, many of the underlying processes that contribute to the disorders of the joints and connective tissue that characterize AA also affect cancer progression and metastasis. Systemically, AA can lead to cellular infiltration and inflammation of the lungs. Several studies have reported statistically significant risk ratios between AA and breast cancer. Despite this knowledge being available, there has been minimal research linking breast cancer, arthritis, and metastasis associated with breast cancer. Notably both diseases are extremely prevalent in older post-menopausal women.

**Methods:**

To establish the novel link between arthritis induced inflammation and secondary metastasis associated with breast cancer, PyV MT mice that spontaneously develop mammary gland carcinoma were injected with Type II collagen (CII) to induce arthritis at 9 and 18 weeks of age for pre-metastatic and metastatic condition. The sites of secondary metastasis and the associated inflammatory microenvironment were evaluated.

**Results:**

A significant increase in breast cancer-associated secondary metastasis to the lungs and bones was observed in the arthritic versus the non-arthritic PyV MT mice along with an increase in primary tumor burden. We report significant increases in the levels of interstitial cellular infiltrates and pro-inflammatory cytokines such as interleukin-17 (IL-17), interleukin-6 (IL-6), Pro- Matrix metallopeptidase 9 (Pro-MMP9), insulin like growth factor-II (GF-II) and macrophage colony stimulating factor (M-CSF) in the arthritic lung and bone milieu as well as in the circulation. These pro-inflammatory cytokines along with the inflammatory microenvironment may be the underlying factors facilitating tumor progression and metastasis in arthritic PyV MT mice. This was further substantiated by treatment with celecoxib, an anti-inflammatory drug + αIL-17 antibody that significantly reduced the secondary metastasis to lung and bone.

**Conclusions:**

The data generated not only reveal the underlying mechanism of high susceptibility to bone and lung metastasis in an arthritic condition but our combination therapies may lead to treatment modalities that will be capable of reducing tumor burden, and preventing relapse and metastasis in arthritic patients with breast cancer.

## Background

While advances have been made in breast cancer therapies, metastatic breast cancer remains an incurable disease, and thus the prevention of metastases must be a priority. The preference of breast cancer cells to grow in the bone and lung is underscored by the fact that 65-75% of patients with advanced disease develop metastasis in these organs [1]. We hypothesize that the pro-inflammatory microenvironment within the bone and lung caused by certain inflammatory conditions may partly account for the high prevalence of secondary metastasis to those organs.

One such common inflammatory condition in humans is autoimmune arthritis (AA) which results in inflammation and deformity of the joints. Other systemic effects associated with arthritis include increased cellular infiltration and inflammation of the lungs [2]. Although AA does not increase the risk for BC, several studies have reported that compared to cancer patients without rheumatoid arthritis (RA), those with RA have poor prognosis and higher mortality. Specifically, patients with non-Hodgkin's lymphoma, skin cancer, and BC have significantly lower survival if they suffer from RA compared to their non-arthritic counterparts [3-8].

Despite this knowledge available for a decade, it has not been fully studied in bones and lungs, the sites of chronic inflammation associated with AA, creates a milieu that attracts tumor cells to home and grow in the inflamed organs which are frequent sites of breast cancer metastasis [8]. There has been minimal research investigating the link between breast cancer-associated metastasis and arthritis even though both diseases share several common molecular pathways of pathogenesis and both diseases are highly prevalent in post menopausal women.

We have recently shown that the incidence of breast cancer-associated bone and lung metastasis was significantly higher in mice that develop spontaneous arthritis[9]. This was the first study that undoubtedly established a correlation between the pro-inflammatory microenvironment in bones and lungs during AA and the homing of circulating tumor cells in these sites of inflammation. Data from these studies were further substantiated in a clinically relevant model of spontaneous metastatic mammary carcinoma induced to develop arthritis. Hence, this study is a sequel of our previous study and our data corroborates a novel link between arthritis induced inflammation and secondary metastasis associated with breast cancer.

The model of spontaneous metastatic mammary gland tumors known as the MMTV-PyV MT mice carry the polyoma virus middle T-antigen driven by the mouse mammary-tumor virus promoter [10-12]. This oncogene is active throughout all stages of mammary gland development, resulting in widespread transformation and production of multifocal mammary adenocarcinomas with ~30-40% of the mice exhibiting lung metastasis by 18-26 weeks of age [10-13]. The PyV MT mice were induced to develop arthritis by administration of Type II Collagen (CII) at two time points: when the mice were 9 or 18 weeks of age designated pre-metastatic or metastatic stage respectively. The collagen-induced arthritis (CIA) model has been the most widely accepted model for inducing AA in mice. CIA is elicited in mice by immunization with CII emulsified in complete Freund's adjuvant (CFA). The ensuing pathogenesis shares several pathological features with rheumatoid arthritis (RA), including synovial hyperplasia, mononuclear cell infiltration, and cartilage degradation and the mechanism by which arthritis is induced by collagen injection in these mice is already established [14,15].

Data clearly demonstrates a significant increase in bone and lung metastasis and decreased survival in the arthritic versus the non-arthritic PyV MT mice. In addition, we have identified some of the key proinflammatory factors in the arthritic lung and bone microenvironment and also in circulation that may contribute to the increased incidence of secondary metastasis. Further, we determined that blocking the COX-2/PGE_2 _and IL-17 pathways significantly reduced the formation of secondary metastasis in the PyV MT mice.

This study is of high importance with vital clinical implications, especially in the prevention of metastasis, in designing combination drug regimens, and as a diagnostic risk-assessment tool in patients with arthritis and breast cancer.

## Methods

### Mice

PyV MT oncogenic mice were originally a gift from Dr. W. J. Muller (McGill University, Toronto, Canada) [10]. The PyV MT mice that we have used are congenic on the C57Bl/6 background and have been used in several of our prior publications [13,16-19]. PCR was used to routinely identify the PyV MT oncogene. PCR was carried out as described previously [13]. Amplification of PyV MT gene results in a 480-bp fragment.

All mice were bred and maintained in specific pathogen-free conditions in the Mayo Clinic Scottsdale Natalie Schafer Transgenic Animal Facility and UNCC Animal Facility. All experimental procedures were conducted according to Institutional Animal Care and Use Committee guidelines. All protocols were approved by the Mayo Clinic and UNCC Internal Animal Care Review Committee.

### Induction of Arthritis

The PyV MT mice with spontaneous breast cancer were injected with 50 μls of 2 mg/ml CII (MD Biosciences, St. Paul, MN, USA) in CFA (Difco laboratories, Michigan, USA) intradermally ~1.5 cms distal from base of tail at two time points, at 9 weeks of age when the primary tumors are undetectable and at 18 weeks of age when the primary tumors are large enough and metastasis is expected to occur [10,11]. Fifty-sixty percent of mice develop arthritis within 15-30 days post collagen injection and the mechanism is established[14].

### Generation of PyV MT cells

To generate the PyV MT cell line, the tumors were dissociated in collagenase IV (1 mg/ml)(Worthington Biochemical Corporation, 730 Vassar Ave, NJ 08701) at 37°C for 30 mins. Then the tumors were meshed, cells counted and ~3 × 10^6 ^cells were plated in C-DMEM. Post 24 hours cells were replenished with fresh media.

### Measurement of PGE_2 _levels in the serum

PGE_2 _levels in the serum were determined as previously reported using a specific ELISA kit for PGE_2 _metabolite (PGEM) (13, 14-dihydro-15-keto-PGA2) (Cayman Chemicals, Ann Arbor, MI, USA) [20]. Manufacturer's recommended protocols were followed. Serum was diluted appropriately to ensure that readings were within the limits of accurate detection. Results are expressed as picograms of PGEM/per ml. of serum.

### Measurement of cytokines

The RayBio^® ^Custom Mouse Cytokines Antibody Array kit was purchased from Ray Biotech (Norcross, GA, USA) and used according to the manufacturer's instructions. To measure the cytokines in the lung and bone microenvironment, 300 and 100 ug of protein was used respectively. Chemiluminescence was detected using an EpiChemi3^® ^Darkroom imaging system and Lab Works^® ^densitometry software (both from UVP Bioimaging, Upland, CA, USA). Data was corrected for background signal and normalized to positive controls using RayBio^® ^Analysis Tool software as published (UVP Bioimaging, Upland, CA, USA).

### Invasion Assays

PyV MT cells were serum-starved for 24 hrs prior to plating for the invasion assay. Cells in serum free media (50,000 cells) were plated over transwell inserts (BD Biosciences, San Jose, CA), pre-coated with reduced growth factor matrigel, and were permitted to invade towards lung lysate (300 ug protein) and bone lysate (100 ug protein) contained in the bottom chamber for 24 hours. Percent invasion was calculated as absorbance of samples/absorbance of controls × 100 [21].

### Histology

Lungs and tumor sections were formalin fixed in 10% neutral-buffered formalin (pH 6.8-7.2) for a minimum of 24 hours. Paraffin embedded blocks was prepared by the Histology Core at The Mayo Clinic and 4-micron thick sections were cut for hematoxylin eosin (H&E) staining and for immuno-staining. Bones were decalcified using Cal-Rite (Richard Allan Scientific, Kalamazoo, MI), a formic acid decalcification agent prior to IHC. For VEGF, Pancytokeratin, and PCNA staining, sections were subjected to antigen retrieval using the DAKO Target Retrieval agent (Dako North America, Carpentaria, CA, USA). Primary antibodies to VEGF, PCNA, and pancytokeratin were purchased from Santa Cruz Biotechnologies, Santa Cruz, CA, USA and BD Biosciences, CA, USA). To determine macrophage infiltration, F4/80 antibody was purchased from Abcam, Cambridge, MA, USA. Corresponding secondary antibodies were purchased from DAKO. For all slides, 3,3" -Diaminobenzidine (Vector laboratories, Burlington, CA, USA) was used as the chromogen and hematoxylin was used as counterstain. For neutrophil staining, a standard Naphthol AS-D Chloroacetate Esterase staining using a kit from Sigma was used. (Sigma, St Louis, MO, USA; Cat: # 91C-1Kit) was used. Masson trichome staining on bone was used to determine levels of osteoclasts. All slides were examined under light microscopy and pictures taken at magnifications indicated in the Figure legend.

### Western Blots and Antibodies

Equal quantities of tumor lysates were loaded on SDS-PAGE gels. COX-2 and β-actin antibodies were purchased from Santa Cruz Biotechnology, Santa Cruz, CA and were used according to manufacturer's recommendations.

### Study design for the IL-17 + celecoxib treatment

To test the efficacy of anti-IL-17 antibody treatment on breast cancer-associated metastasis, PyV MT mice were injected with 50 μls of 2 mg/ml CII in CFA at 12 wks. Post 3-weeks of collagen injection, four i.p injections of 5 μg/ml of anti-IL17 antibody (BD Pharmingen, San Diego, CA) once every two weeks was administered. Celecoxib (20 mg/kg in 100 μl 10% DMSO) was gavaged [22] starting at the same time as the anti-IL-17 antibody but was given daily until sacrifice except for the weekends. One week after the last injection, mice were sacrificed. Controls included PyV MT mice induced with arthritis and injected with 5 μg/ml rat immunoglobulin (Ig) G1 control antibody (BD Pharmingen, San Diego, CA) in 100 μl PBS.

### Image Acquisition and Analysis

Bright field images were captured from immunostains of the mouse tissue using an Olympus microscope Olympus BX60, U-ND25-2 (Olympus; Melville, NY) with magnifications (referred in Figure Legend) using the DP70 controller, processor and analysis software.

Red-green-blue (RGB)-filtered grayscale values from images from microscopic slides of mouse tissue, stained with DAB, hematoxylin or both were analyzed using the Image-Pro Plus and NIH Image processing and analyzing program [22]. A simple translation algorithm using the RGB information was developed, providing the option for separation of DAB only- and double-stained areas from hematoxylin only-stained areas by means of subject specific thresholding, based on the correlations between the R-G- and B-filtered grayscale values. A good separation of DAB- and double-stained pixels from hematoxylin-stained pixels was achieved. Significant differences in relative areas stained and mean specific intensity for the stains between control and treatment groups in mouse tissue were tabulated. N = 3 mice and 5 fields are provided.

The densitometric analyses of immunoblots were performed using NIH Image (obtained from the NIH Web site: http://rsb.info.nih.gov/nih-image). Results are presented as mean values of arbitrary densitometric units corrected for background intensity and normalized to the expression of β-actin, or as fold increase over levels in unstimulated cells.

### X-Ray imaging

The Pix array 100 x-ray machine was used for bone imaging. The Pix array 100 (Bi Optic Inc, Santa Clara, CA, USA) is a commercially available x-ray machine that is designed for animal x-ray[23]. The analysis was conducted in Carolinas Medical Center within the Department of Orthopedic Surgery.

### Statistical analysis

Data were analyzed using GraphPad software (GraphPad Prism version 4.00 for Windows; GraphPad Software, San Diego, CA, http://www.graphpad.com). Results expressed as mean ± SEM and are representative of greater than or equal to three separate experiments. Comparison of groups were performed using one-way or two-way ANOVA followed by the Bonferroni post-test for multiple comparisons (*p < 0.05, **p < 0.01, ***p < 0.001). Student's t-test was used for comparing the level of significance between the experimental groups.

## Results

### Decreased survival in arthritic versus non-arthritic PyV MT mice

**Figure 1:**
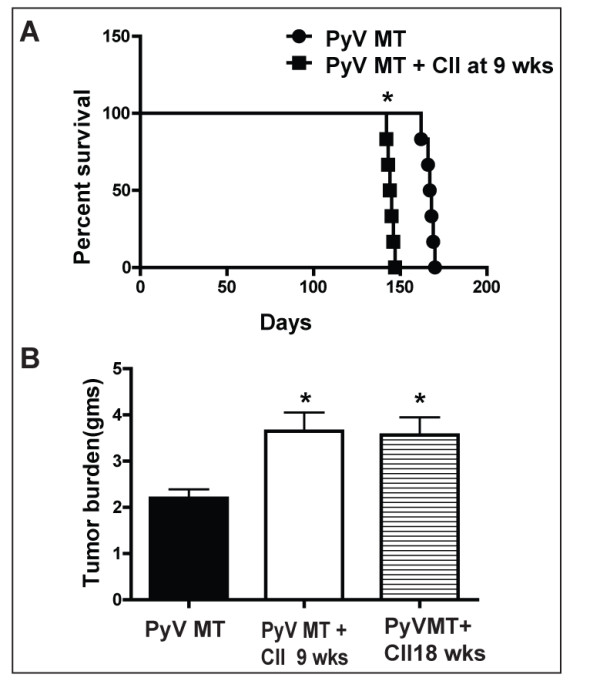
**Lower survival coupled with higher tumor burden of the arthritic versus non-arthritic PyV MT mice**. **A: **Significant diminution in the survival of arthritic versus non-arthritic PyV MT mice. N = 6 mice [*p < 0.01 (CII injected at 9 wks of age)]. **B: **Significantly higher tumor burden in arthritic versus non- arthritic PyV MT mice. N = 10 mice. *p < 0.01 (CII injected at 9 or 18 weeks) compared to non-arthritic PyV MT mice.

Survival was assessed in PyV MT mice that were induced to develop autoimmune arthritis (AA) with collagen II injection at week 9 week of age. The PyV MT mice develop hyperplasia when the mice hit puberty around 6-8 weeks of age followed by carcinoma-in-situ and palpable mammary gland tumors by 12-14 weeks of age leading to invasive adenocarcinoma by 18-24 week of age. Thus, we were able to study the effect of arthritis on survival when AA was induced at the pre- metastatic stages (at 9 weeks of age). This model is clinically relevant, as tumors arise in an appropriate microenvironment, in the context of a viable immune system, and are phenotypcially similar to human breast tumors. The survival of the PyV MT mice was significantly diminished with collagen-induced arthritis where all arthritic mice had to be euthanized by 149 days (~ 21 weeks) due to high tumor burden, ulceration of tumor, sluggish motion, hunched back and interferences with normal ambulation compared to 170+days (~24-25 weeks) for PyV MT mice without arthritis (P < 0.001)(Figure 1A).

### Remodeling of the primary mammary gland tumor in arthritic PyV MT mice

**Figure 2:**
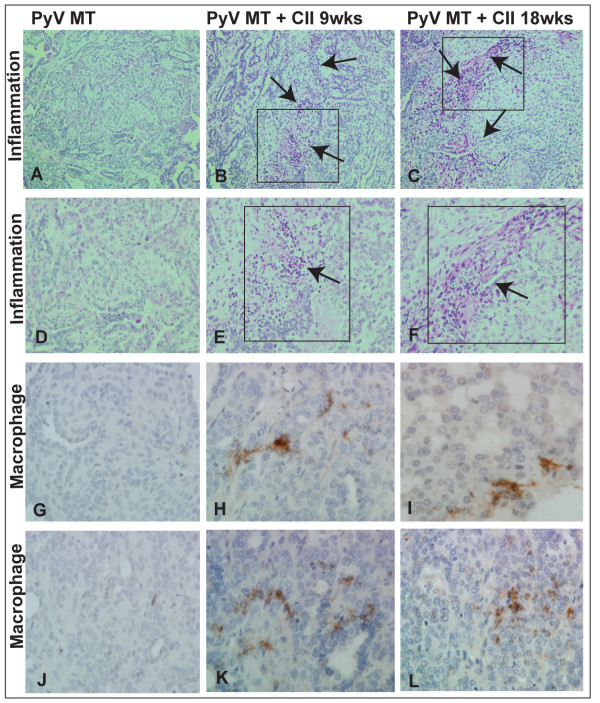
**Increased cellular infiltration within the PyV MT tumors of arthritic versus non-arthritic mice**. **A - F: **H&E staining showing inflammation in the tumor microenvironment; **A and D: **PyV MT (Low inflammation); **B and E**: PyV MT + CII at 9 (severe inflammation); C and F: PyV MT + CII at 18 wks (severe inflammation). The images of tumor sections were taken at 200× (**A-C**) and 400× (**D-F**) magnification from the same area. Representative of n = 10 mice is shown. **G-L**: Increased macrophage infiltration within the PyV MT tumors of arthritic versus non-arthritic mice indicated by F4/80 staining. The images of the macrophage staining were taken at 600× magnification. Representative of n = 3 mice is shown.

PyV MT mice were induced to develop autoimmune arthritis with collagen II injections at week 9 and week 18 of age (pre and post metastatic stage). We questioned if the primary tumor itself was affected by the arthritic milieu. The primary tumor burden was significantly increased in the PyV MT mice with arthritis compared to PyV MT mice without arthritis (P < 0.01, n = 10-13 mice) (Figure 1B) regardless of whether arthritis was induced at pre or post-metastatic stage. Higher tumor burden correlated with increased cellular infiltration within the tumor microenvironment which was determined by quantifying the areas of infiltration in the H&E stained tumor sections (Figure 2A-F).

**Figure 3:**
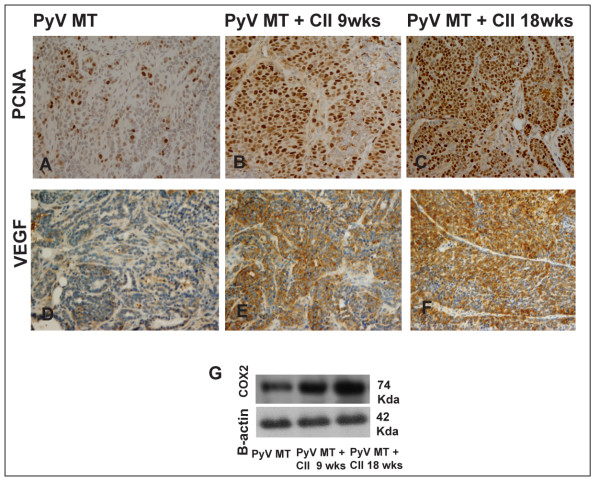
**Increased expression of PCNA, VEGF and COX-2 in the arthritic PyV MT tumors**. **A-C**: Higher number of PCNA positive cells in the tumors of arthritic PyV MT versus non-arthritic PyV MT. **D-F: **Increased expression of VEGF in the arthritic PyV MT tumors. The images of tumors were taken at 200× magnification. Brown staining represents positive PCNA and VEGF staining. Representative of n = 10 mice is shown. **G: **Western blot analysis of COX-2 expression. β-actin serves as control for equal protein loading.

Integrated density was used to quantify the levels of infiltrating cells. Quantification was based on 5 fields with n = 3 tumor sections per experimental group and presented in Table 1. Further, we show increased macrophage infiltration within the PyV MT tumors of arthritic versus non-arthritic mice indicated by F4/80 staining (Figure 2G-L). The number of F4/80 positive cells have been counted in 5 fields in n = 3 tumor sections from each experimental group and results documented in Table 2. This was accompanied by increased levels of proliferating cell nuclear antigen (PCNA) staining (Figure 3A-C) within the tumor implying higher proliferation in the arthritic versus the non-arthritic tumors. Table 3 shows the number of PCNA-positive cells in 5 sections in n = 3 tumors from each experimental group. Since cyclooxygenase-2 (COX-2) and vascular endothelial growth factor (VEGF) are hallmarks of inflammation, angiogenesis, and metastasis, we investigated the expression of COX-2 and VEGF in the tumors of our experimental mice. Western blotting was used to determine COX-2 levels and IHC used to determine VEGF levels. Significant increases in VEGF and COX-2 expression was detected in the primary tumors of the arthritic versus the non-arthritis PyV MT mice (Figure 3D-F and Figure 3G). IHC and Western blots were quantified and results reported in Tables 4 and 5. Data suggests that the induction of AA in PyV MT mice creates a pro-inflammatory and angiogenic microenvironment in the primary tumor, further promoting tumor progression. All IHC staining were quantified using the Image-Pro Plus and NIH Image processing and analysis programs.

**Table 1:** Quantification of inflammation in the tumors of arthritic versus non-arthritic PyV MT mice.

Quantification in tumor	IntDen Inflammation
PyV MT	50.3 ± 19
PyV MT + CII at 9 wks	322 ± 17
PyV MT + CII at 18 wks	337 ± 22

Integrated density (IntDen) from 5 fields per tumor in n = 3 animals from each experimental group.

**Table 2:** F4/80 positive cells in the tumors of arthritic versus non-arthritic mice (5 fields from n = 3 mice were counted).

	F4/80 positive cells at 600× magnification						
	**Field 1**	**Field 2**	**Field 3**	**Field 4**	**Field 5**	**Average**	**StdDev**

PyV MT 1	1	1	2	0	0	0.8	0.84

PyV MT 2	0	0	4	1	1	1.2	1.64

PyV MT 3	3	1	2	0	0	1.2	1.30

PyV MT + CII at 9 wks 1	23	15	12	4	5	11.8	7.79

PyV MT + CII at 9 wks 2	19	7	10	18	17	14.2	5.36

PyV MT + CII at 9 wks 3	22	16	18	21	16	18.6	2.79

PyV MT + CII at 18 wks 1	23	15	19	14	16	17.4	3.65

PyV MT + CII at 18 wks 2	18	18	12	13	22	16.6	4.10

PyV MT + CII at 18 wks 3	17	23	15	19	17	18.2	3.03

**Table 3:** PCNA positive cells in the tumors of arthritic versus non-arthritic mice (5 fields from n = 3 mice were counted).

	PCNA positive cells at 400× magnification						
	**Field 1**	**Field 2**	**Field 3**	**Field 4**	**Field 5**	**Average**	**StdDev**

PyV MT 1	50	30	34	50	55	43.8	11.05

PyV MT 2	27	40	50	25	32	34.8	10.28

PyV MT 3	34	37	48	53	50	44.4	8.38

PyV MT + CII at 9 wks 1	100	87	92	95	84	91.6	6.35

PyV MT + CII at 9 wks 2	105	94	86	96	107	97.6	8.56

PyV MT + CII at 9 wks 3	97	80	78	94	73	84.4	10.50

PyV MT + CII at 18 wks 1	120	80	125	115	85	105	20.92

PyV MT + CII at 18 wks 2	97	112	87	88	94	95.6	10.06

PyV MT + CII at 18 wks 3	89	75	96	74	85	83.8	9.36

**Table 4:** Quantification of VEGF staining in tumors (Average IntDen from 5 fields per tumor from n = 3 mice is shown).

Quantification in tumor	IntDen
	**VEGF**

PyV MT	424.93 ± 19

PyV MT + CII at 9 wks	2021.8 ± 21

PyV MT + CII at 18 wks	2788.8 ± 28

**Table 5:** Densitometry analysis of the COX-2 expression in the tumor using the image J software **(**data from n = 3 mice is shown)

Groups	Densitometry analysis: COX2
PyV MT 1	22.28

PyV MT 2	24.21

PyV MT 3	21.67

PyV MT + CII at 9 wks 1	41.65

PyV MT + CII at 9 wks 2	40.45

PyV MT + CII at 9 wks 3	50.23

PyV MT + CII at 18 wks 1	58.17

PyV MT + CII at 18 wks 2	55.41

PyV MT + CII at 18 wks 3	45.15

### Significant increase in osteolytic metastatic lesions in the arthritic PyV MT versus non-arthritic PyV MT mice

**Figure 4:**
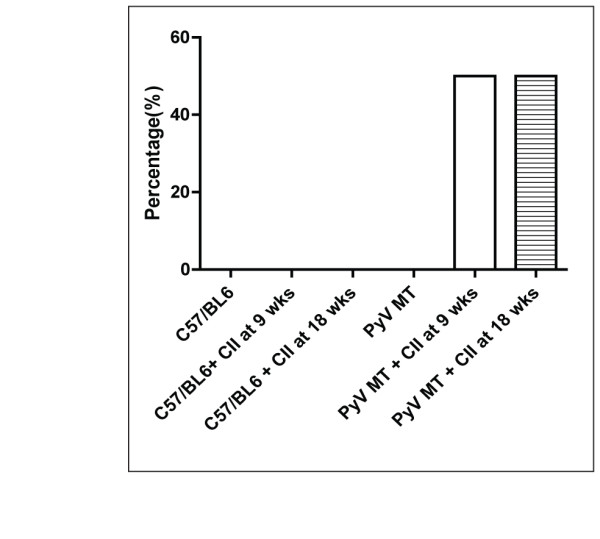
**Percentage of mice with bone metastasis**. Fifty percent of arthritic PyV MT mice developed bone metastasis compared to its non-arthritic counterpart with no bone metastasis (n = 10-13 mice).

**Figure 5:**
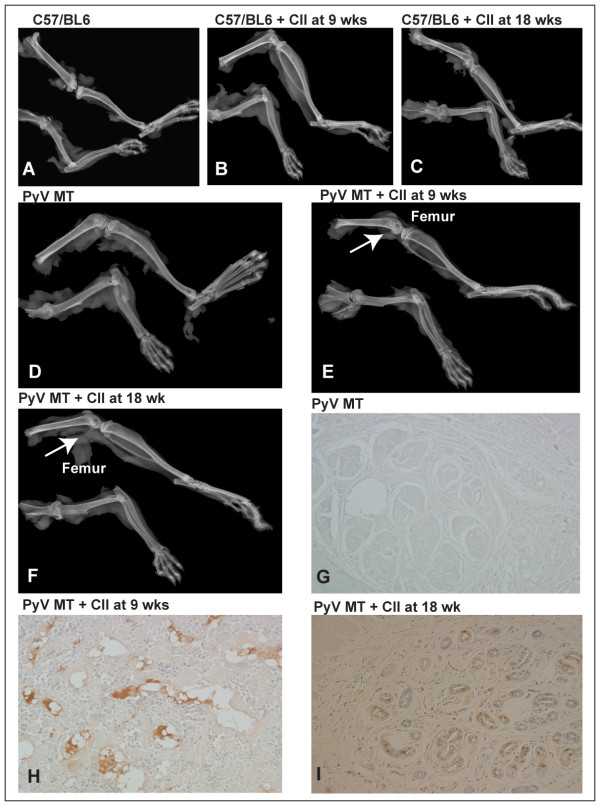
**Metastatic bone lesions accompanied with increased expression of Pancytokeratin in the bones of arthritic PyV MT mice**. **A-F: **Representative x-ray images of bones. **A-D: **Bones from C57/BL6 mice injected with CII at 9 and 18 wks and PyV MT mice with no metastatic lesions; **E and F**: Bones from PyV MT + CII at 9 wks and 18 wks respectively (shows distinct severe osteolytic bone lesion in distal femur as indicated by the arrow). N = 8 mice from each group were examined using the x-ray imaging. **G-I: **Pancytokeratin positive staining observed in the bones of arthritic PyV MT mice. Brown staining represents positive staining. The images were taken at 400× magnification. Representative of n = 10 mice is shown.

We observed that 50% of arthritic PyV MT mice developed bone metastasis while none of the non-arthritic PyV MT mice showed bone metastasis (Figure 4). Bones from n = 8 mice were analyzed by x-ray imaging for osteolytic lesions. Representative images from these groups are shown in Figure 5A-F. Clear osteolytic lesions were evident in the femur of the arthritic but not the non-arthritic PyV MT bones (as indicated by the arrow). The control C57BL/6 mice induced with arthritis did not show any lesions which confirms that the osteolytic lesions are driven by the primary mammary gland tumors.

To further confirm epithelial cell lesions, the bone sections were stained with pancytokeratin and results are shown in Figure 5G-I. Clear lesions are detected in the arthritic PyV MT bones but not in the non-arthritic PyV MT bones.

### Significant increase in lung metastasis in the arthritic PyV MT mice

**Figure 6:**
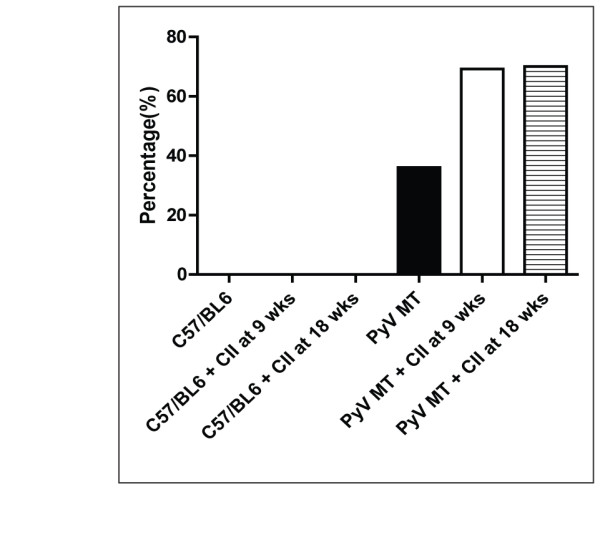
**Percentage of PyV MT mice that developed lung metastasis**. PyV MT mice + CII at 9 and 18 wks had two fold increase in lung metastasis as compared to PyV MT without CII (n = 10-13 mice).

**Figure 7:**
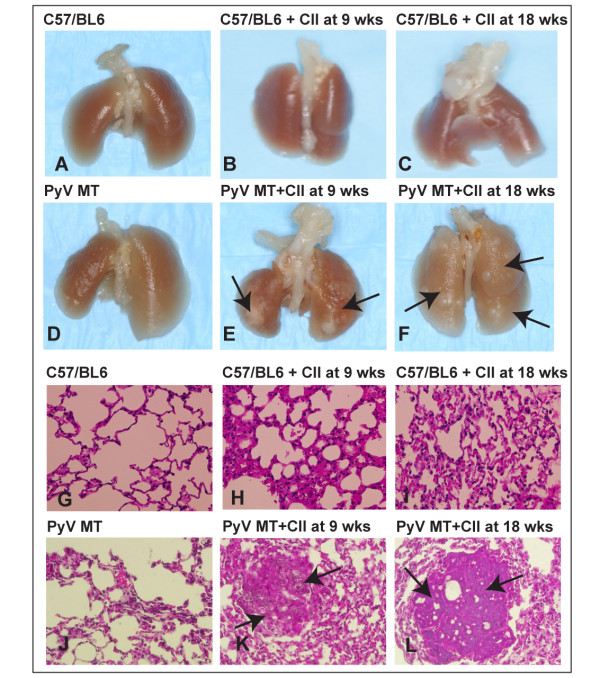
**Images demonstrating clear metastatic lesions in the lungs of arthritic PyV MT mice**. **A-F: **Representative images of lungs showing metastatic lesions. **A-C:**C57/BL6 mice, C57/BL6+CII at 9 and 18 wks (no metastatic lesions) **D**: PyV MT mice(no metastatic lesions); **E and F: **PyV MT+ CII at 9 and 18 wks respectively (arrows represent lung lesions); **G-L: **H&E of the lung sections: The arrows represent metastatic lesions in PyV MT + CII at 9 and 18 wks (K and L) versus no lesions in control C57/BL6, C57/BL6 + CII at 9 or 18 wks and PyV MT lung (G - J). N = 10-13 mice from each group were examined with similar results. The images of the H&E sections were taken at 400× magnification.

We observed >2-fold increase in the incidence of lung metastasis in the arthritic versus non-arthritic PyV MT mice (7/10 mice with CII administered at 18 wks, and 9/13 mice with CII injected at 9 wks) compared to the PyV MT mice with no CII (3/11 mice) (Figure 6). Lung lesions were visualized under dissecting microscope as indicated by arrows (Figure 7A-F) and by H&E staining and histology showing clear metastasis (indicated by arrows) (Figure 7G-L). These results are especially significant since it represents true metastasis arising from the spontaneously occurring primary mammary gland tumors.

### Significant inflammation detected in the bones and lungs of arthritic PyV MT mice

**Figure 8:**
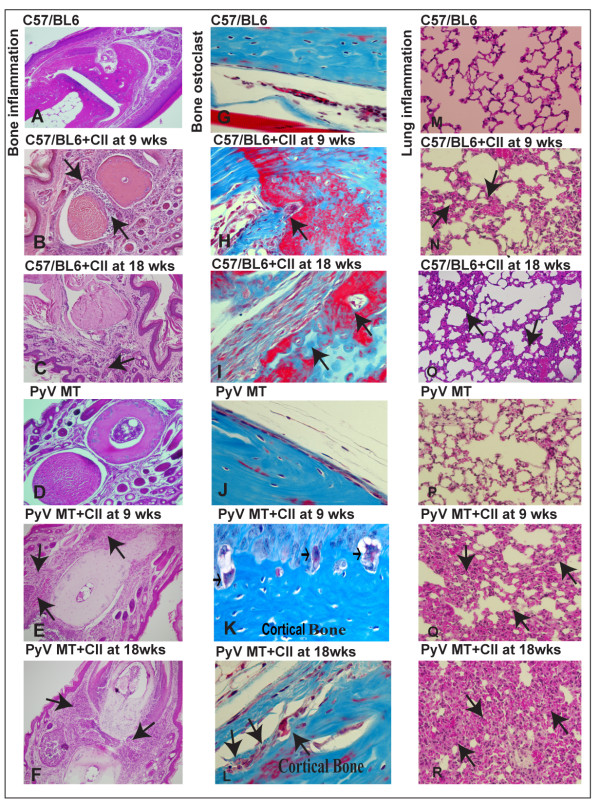
**Histology of bones and lungs, and osteoclast staining of bones of the arthritic versus non-arthritic PyV MT mice**. **A-F: **H&E staining of sections from the joints. **A: **C57BL/6 (no inflammation)**; B: **C57BL/6 mice + CII at 9 wks (moderate inflammation); **C: **C57BL/6 mice + CII at 18 wks (moderate inflammation); **D: **control PyV MT mice (no inflammation); **E: **PyV MT mice + CII at 9 wks (erosion of articular cartilage and bone with severe inflammation); **F**: PyV MT mice + CII at 18 wks (severe inflammation in the phalangeal joints); **G-L: **Masson trichome staining of bone sections for presence of osteoclasts. **G: **C57/BL6 mice (no osteoclasts); **H: **C57BL/6 + CII at 9 wks (few osteoclasts marked with arrows); **I: **C57BL/6 mice + CII at 18 wks (few osteoclasts marked with arrows); **J: **PyV MT mice (no osteoclasts); **K**: PyV MT mice + CII at 9 wks (multiple osteoclasts marked with arrows); **L: **PyV MT mice + CII at 18 wks (multiple osteoclasts marked with arrows). **M-R: **H&E staining of sections from the lungs. **M: **control C57BL/6 (no inflammation); **N: **C57BL/6+CII at 9 wks(moderate inflammation); **O: **C57BL/6+CII at 18 wks(moderate inflammation); **P**: control PyV MT mice (no inflammation); **Q: **PyV MT mice + CII at 9 wks (severe inflammation); **R: **PyV MT mice + CII at 18 wks (severe inflammation). The H&E images of the joints and lungs were taken at 200× and 400× magnification respectively and the Masson Trichome bone images taken at 400× magnification. Representative of n = 10 mice is shown.

To decipher why primary tumor cells are attracted to the arthritic bones, we initially conducted histology of bone sections from all six experimental groups. Representative images of H&E staining are shown in Figure 8A-F for bone sections from C57/BL6, C57BL/6 + CII at 9 wks, C57BL/6 + CII at 18 wks, PyV MT, PyV MT + CII at 9 wks, and PyV MT + CII at 18 wks. Enhanced inflammation with increased cellular infiltration was clearly observed in the C57/BL6 bones from arthritic mice as compared to the non-arthritic C57BL/6 and PyV MT bones. The severity of inflammation was augmented with arthritic PyV MT bones suggesting that the metastatic PyV MT tumor may have the potential to enhance the severity of arthritis (Figure 8A-F). N = 8 mice were evaluated with similar results. The results are tabulated as integrated density (IntDen) from n = 3 mice in Table 6. Inflammatory signals are known to induce osteoclast maturation and bone resorption during CII-induced arthritis. Such phenomena mainly occur at the interface between proliferating synovium and bone tissue in arthritis. High cellular infiltration in the arthritic PyV MT mice was associated with increased bone destruction as evidenced by the increased osteoclasts in these mice (Figure 8G-L) as compared with PyV MT with no CII. Taken together these data suggest that the metastatic breast cancer cells may contribute to the vicious cycle of osteolytic destruction.

**Table 6:** Quantification of inflammation in the bones (Average IntDen from 5 fields per bone from n = 3 mice is shown).

Quantification of inflammation in bone	
**Group**	**IntDen**

C57BL/6	0

C57BL/6 + CII at 9 wks	50 ± 38

C57BL/6 + CII at 18 wks	72 ± 12

PyVMT	0.0

PyV MT +CII at 9 wks	232 ± 15

PyV MT +CII at 18 wks	265 ± 20

To further demonstrate the chemotactic microenvironment in the lungs of arthritic versus non-arthritic mice, lung histology was examined. Moderate inflammation was noted in the C57BL/6 mice with arthritis compared to no inflammation in the non-arthritic C57BL/6 lungs. Significantly enhanced inflammation with increased cellular infiltration was observed in the lungs of PyV MT mice injected with collagen compared to PyV MT mice without collagen and compared to control C57BL/6 mice with collagen (Figure 8M-R). The pro-inflammatory phenotype in the lung correlated with the severity and incidence of lung metastasis (Figure 7) suggesting the critical role of inflammatory cells in promoting metastasis.

**Figure 9:**
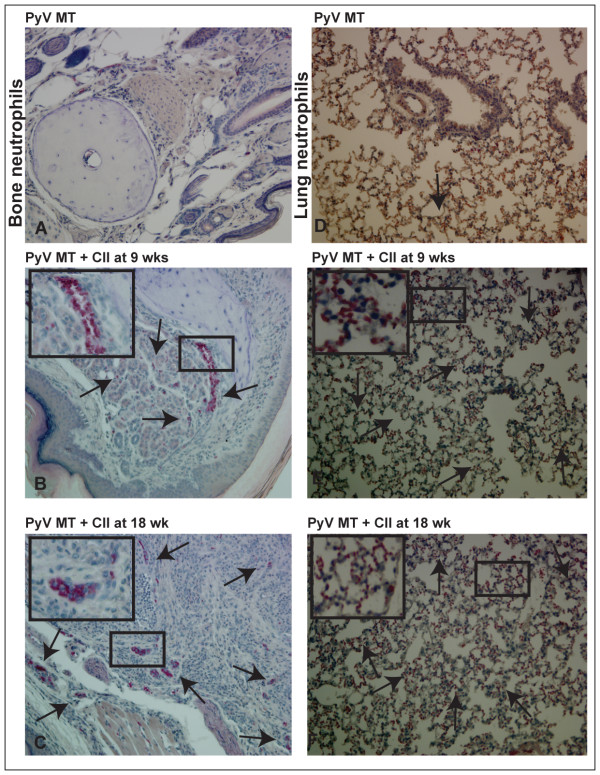
**Increased infiltration of neutrophils in the bones and lungs of arthritic PyV MT mice**. **A-F: **Naphthol AS-D chloroacetate esterase staining of the bones and lungs for neutrophils. Increased infiltration of neutrophils in **bones (A-C) and lungs (D-F) **of arthritic PyV MT mice indicated by arrows. Representative of 10 fields in n = 10 mice is shown. The images of the bones and lungs were taken at 400× and 200× magnification respectively.

In addition, we demonstrate neutrophillic infiltration in the bones and lungs of arthritic versus non-arthritic PyV MT mice, another indicator of increased inflammation in the arthritic organs. Representative images are shown in Figure 9A-C for bones and Figure 9D-F for lungs from the arthritic and non-arthritic PyV MT mice.

### Enhanced invasion of PyV MT tumor cells towards arthritic bone and lung lysate

**Figure 10:**
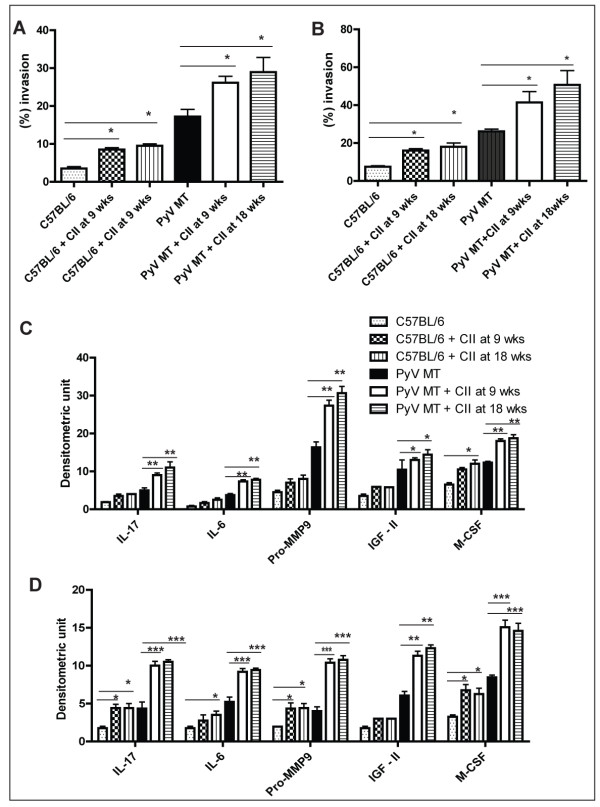
**Higher invasion of PyV MT cells towards the bone and lung lysate of the arthritic versus non-arthritic PyV MT mice**. Up regulation of several cytokines in the lung and tumor microenvironment. **A and B: **Significantly higher invasion index of the PyV MT tumor cells towards the arthritic bone and lung lysate in an *in vitro *matrigel invasion assay (* p < 0.05 compared to the non-arthritic PyV MT lung and bone lysate). **C and D: **A graphical representation of the up regulated cytokines in the bone and lung lysate (*p < 0.05, **p < 0.01, ***p < 0.001). The Ray Biotech cytokine array kit was utilized for this experiment. Average of n = 4 mice. The statistical differences between groups are shown with horizontal bars. We have compared 1) C57Bl/6 with C57Bl/6 + CII at 9 and 18 weeks; and 2) PyV MT with PyV MT + CII at 9 and 18 weeks.

Thus far, our data suggests that the increased cellular infiltration in the lungs and bones of the arthritic mice versus the non-arthritic mice may be one of the underlying mechanisms for the increased rate of metastasis observe in the arthritic mice (Figures 4, 5, 6, 7, 8, 9). To substantiate the chemotactic potential of the arthritic bone and lung, bone and lung lysates of the arthritic and non-arthritic mice were used as the chemotactic factor in an *in vitro *trans-well matrigel invasion assay with the PyV MT cells (generated from primary PyV MT tumors) in the top chamber and the bone and lung lysates in the bottom chamber. Data clearly shows that the lung and bone microenvironment was significantly altered in the arthritic mice to become more chemo-attractant to the PyV MT tumor cells (Figure 10A and 10B). Statistically significant difference is provided between PyV MT and PyV MT + CII at 9 and 18 weeks as well as C57Bl/6 and C57Bl/6 + CII at 9 and 18 weeks.

### IL-17, IL-6, Pro-MMP9, IGF-II, and M-CSF may be the underlying factors responsible for the increased metastasis in the lungs and bones of arthritic mice

To determine which factors in the bone and lung microenvironment may be responsible for higher invasion, thereby driving the breast cancer cells to become more metastatic in the arthritic model, we used the RayBio^® ^Custom Mouse Cytokines Antibody Array. The arthritic lungs and bones expressed significantly higher levels of cytokines and growth factors which included IL-17, IL-6, Pro-MMP9, IGF-II, and M-CSF (Figure 10C and 10D). This was regardless of whether the arthritis was induced at 9 or 18 wks of age suggesting that the arthritic milieu remains stable even at 10-12 weeks post CII injection. The levels of the pro-inflammatory cytokines were found to be higher in arthritic C57BL/6 lungs and bones compared to the non-arthritic C57BL/6.

Thus, we hypothesize that the pro-inflammatory microenvironment in the arthritic bone and lungs may boost the recruitment of the PyV MT tumor and that the PyV MT tumor in turn significantly augments the levels of the cytokines in these target organs thus creating a highly conducive microenvironment for the PyV MT tumors to further proliferate.

### High levels of circulating PGE_2 _coupled with increased levels of pro-inflammatory cytokines in circulation may initiate primary tumors to be more metastatic in arthritic milieu

**Figure 11:**
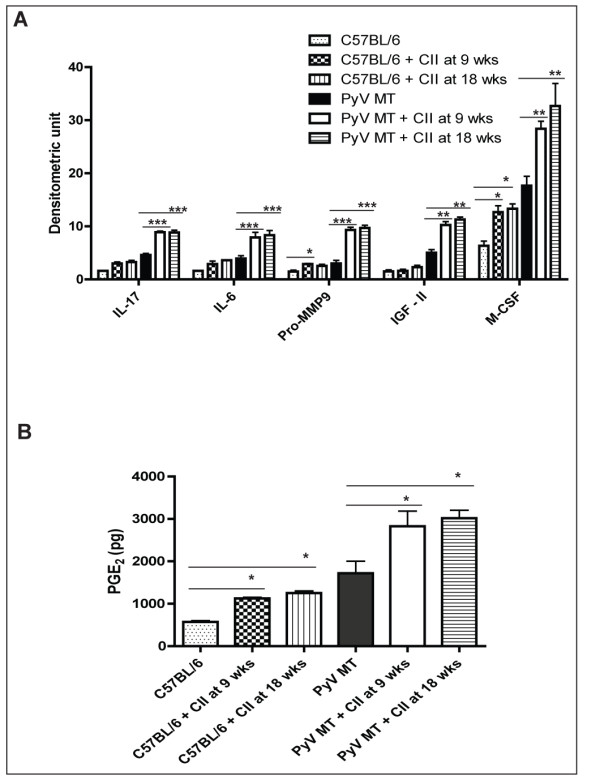
**Serum analysis revealed up regulation of various cytokines along with PGE2 which may be the underlying factor contributing to increased metastasis in arthritic mice**. **A: **A graphical representation of the up regulated cytokines in the bone and lung lysate (*p < 0.05, **p < 0.01, ***p < 0.001). The Ray Biotech cytokine array kit was utilized for this experiment and average of n = 4 mice. **B: **Increased circulating PGE_2 _in the arthritic versus the non-arthritic PyV MT mice (*p < 0.01 versus PyV MT mice), n = 10 mice per group. A specific ELISA was used for this experiment. The statistical differences between groups are shown with horizontal bars. We have compared the statistical differences between 1) C57Bl/6 with C57Bl/6 + CII at 9 and 18 weeks; and 2) PyV MT with PyV MT + CII at 9 and 18 weeks.

We also evaluated the circulating levels of pro-inflammatory cytokines and chemokines in the sera of the arthritic versus the non-arthritic mice. These same factors (IL-17, IL-6, Pro-MMP9, IGF-II, and M-CSF) were also found to be elevated in the circulation suggesting their role in possibly initiating the primary tumors to be more metastatic (Figure 11A). Data is presented as densitometry units.

Lastly, but expectedly, we detected significant increase in PGE_2 _levels in the circulation (Figure 11B). Elevated PGE_2 _is a hall mark of arthritis and is known to enhance primary tumor cells to become highly angiogenic and metastatic.

### Treatment with anti-IL-17 and a COX-2 inhibitor significantly reduced the secondary metastasis in the arthritic PyV MT mice

**Figure 12:**
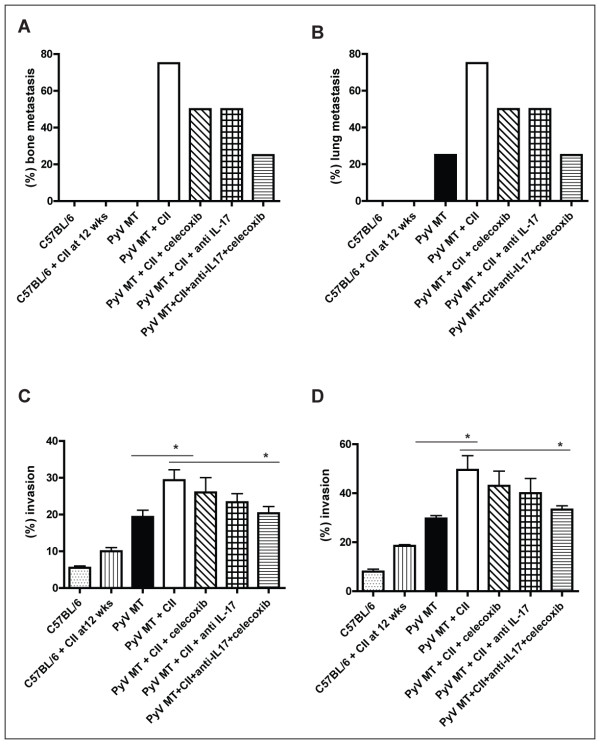
**Treatment with anti-IL17 antibody and celecoxib significantly reduced secondary metastasis in the arthritic PyV MT mice**.Percent of mice that developed **A) **bone and **B) **lung metastasis post treatment. Fifty percent decrease in bone and lung metastasis in mice treated with the combination therapy. N = 4 mice per group. **C and D: **Percent of PyV MT cells that invaded through the matrigel in an *in vitro *invasion assay with **C) **bone lysate or **D) **lung lysate in the bottom chamber. Lysates from lung and bone of treated arthritic PyV MT mice were significantly less chemotactic than lysates from untreated arthritic PyV MT mice. (* p < 0.05). N = 3 from each group. The statistical differences between groups are shown with horizontal bars. The statistical differences were compared between 1) C57Bl/6 with C57Bl/6 + CII at 9 and 18 weeks; and 2) PyV MT with PyV MT + CII at 9 and 18 weeks.

The goal of our studies is to find a possible therapy for arthritis-induced breast cancer metastases. Both IL-17 and COX-2 are reasonable targets as both were up-regulated in the arthritic mice and both are used clinically for treatment of arthritis. IL-17 is known to also mediate proinflammatory effects by stimulating the release of multiple other cytokines such as IL-6, IL-8, GM-CSF, TGF-β, TNF-α and G-CSFs from epithelial, endothelial, and fibroblastic cells. In addition, it is an emerging therapeutic target for cancer metastasis and arthritis [24-26]. High levels of cyclooxygenase 2 (COX-2) is linked to both AA and breast cancer metastasis [27]. We treated the arthritic PyV MT mice with a combination of celecoxib, a specific COX-2/PGE_2 _inhibitor, and a neutralizing antibody against IL-17. Excitingly, the incidence of secondary metastasis was significantly reduced in the arthritic PyV MT mice treated with a combination of celecoxib and a neutralizing antibody against IL-17 (Figure 12A and 12B). Lysates from metastatic sites in treated mice were further evaluated for their chemo attractant properties and were found to be significantly less attractant than bone and lung lysates from untreated arthritic PyV MT mice (Figure 12C and 12D). In fact the level of chemo attractant was as low as the non-arthritic PyV MT mice suggesting that the anti-IL-17 antibody and celecoxib reduced the levels of IL-17 and PGE_2 _which in turn reduced the levels of all other factors that were up regulated in the arthritic lung and bone lysates (data not shown).

## Discussion

Inflammation is a critical component of tumor progression and metastasis as well as arthritis [28-31]. Many processes that occur during arthritis also occur during tumorigenesis including uncontrollable growth, inflammation, increased vascularity and common cytokines and growth factors that are regulated in both. The tumor microenvironment, which is largely orchestrated by inflammatory cells, is critical in the neoplastic process, fostering proliferation, survival and migration [32]. It is indeed interesting that post-menopausal women who are usually prone to developing some form of autoimmune arthritis including osteoarthritis, RA, or inflammatory polyarthritis are also the most likely candidates to develop breast cancer. Thus, it is not unlikely that the two diseases co-exist in these women. Although there are several studies implicating inflammation as the initiator of tumor formation and/or mediator of progression, there is minimal research on whether prior inflammation at the site of metastasis produces a fertile ground for primary tumor cells to home and proliferate. Our study begins to evaluate whether arthritis which causes inflammation in the bones and lungs enhances secondary metastasis to those sites. A significant increase in breast cancer-associated metastasis to the lungs and bones was observed in the arthritic versus the non-arthritic PyV MT mice (Figures 4, 5, 6, 7) along with increase in primary tumor burden (Figure 1). Compared with the non-arthritic C57BL/6, the lungs and bones of the arthritic C57BL/6 expresses moderate levels of inflammation even before any tumor challenge suggesting a pro-inflammatory milieu that may be responsible for attracting the PyV MT metastatic cells to the lungs and bones as demonstrated in the *in vitro *invasion assay (Figure 10A and 10B). Once the PyV MT cells home to the lungs or bones, the levels of interstitial cellular infiltrates and pro-inflammatory cytokines are exponentially increased which is characterized by prominent cytokines IL-17, IL-6, Pro-MMP9, IGF-II and M-CSF (Figure 10C and 10D). The levels of these cytokines along with PGE_2 _are also upregulated in the circulation (Figure 11A and 11B). These pro-inflammatory cytokines along with inflammatory microenvironment can facilitate tumor cell extravasation and promote metastasis. This is further substantiated when treatment with celecoxib + αIL-17 antibody significantly reduced the metastasis to lung and bone (Figure 12). This study undoubtedly authenticates our previous study [9].

The tumor microenvironment itself is regarded as a "smoldering" inflammation site in which many cytokines, chemokines, and enzymes mediate the inflammatory process and drive malignant progression [33]. We observed increased inflammation into the tumor site of the arthritic PyV MT mice possibly remodeling the tumor microenvironment (Figure 2). For tumors to develop in size and amplify metastatic potential, they must make an "angiogenic switch" through perturbing the local balance of pro-angiogenic and anti-angiogenic factors. Frequently, tumors over express pro-angiogenic factors, such as PGE_2 _and VEGF, allowing them to make this angiogenic switch [34]. We observed increased VEGF and COX-2 expression in the tumors of arthritic PyV MT mice compared to non-arthritic tumors (Figure 3) possibly inducing an "angiogenic switch" and contributing to invasiveness of the cells.

Cytokines and prostaglandins play an essential role in the development of arthritis [35]. Several cytokines have been implicated in the mechanism of synovial cell activation and joint destruction in AA [36]. At the same time, cytokines also play an imperative role in cancer development and progression. In fact, elevated serum M-CSF predicts reduced survival in metastatic breast cancer patients [37]. At the same time, the M-CSF produced by breast cancer cells and surrounding stroma increases osteoclast formation and maturation and enhances the expression of stromal RANK ligand, both of which increase osteolytic bone degradation [38]. M-CSF also contributes to the pathogenesis of RA through up regulation of neutrophil gelatinase-associated lipocalin (NGAL) in neutrophils, followed by induction of transitional endoplasmic reticulum ATPase (TERA), cathepsin D and transglutaminase 2(TG2) in synoviocytes [39]. Pro-MMP9 concentration in sera and joint fluids of RA patients is reported to be significantly higher which correlates with our mouse model where the Pro-MMP9 levels are up regulated in the arthritic bone, lungs microenvironment as well as in the sera [40]. It is reported that cathepsin G is up regulated through tumor stromal interactions and activates Pro-MMP9, active MMP9 cleaves and releases active TGF-beta, and active TGF-beta can then promote tumor growth and enhance osteoclast activation and subsequent bone resorption [41]. Over expression of IGF-II is reported in multiple types of cancer and is proposed as a potential mechanism for cancer cells to develop resistance to IGF-1R-targeting therapy [42]. IL-17 acts on osteoblasts by stimulating COX-2-dependent PGE_2 _and osteoclast differentiation factor which differentiates osteoclast progenitors into mature osteoclasts, causing bone resorption. PGE_2 _interacts with its eicosanoid receptors to induce the damage [26]. It is found that synovial fluids of patients with RA contain high levels of the cytokines IL-17 and IL-15 [43]. Cytokines play a crucial role in the regulation of inflammatory events. Inflammatory disorders such as RA are characterized by an overproduction of several cytokines including IL-6 [44]. IL-6 on the other hand is an autocrine and paracrine growth factor for several cancers, including breast cancer [45,46] and both IL-17 and IL-6 stimulates cancer cell growth and contributes to recurrence and metastasis in breast cancer [47-49].

## Conclusion

The data clearly shows that breast cancer associated metastasis is increased in arthritic conditions and blocking the IL-17 and COX-2 pathways significantly reduces the development of secondary metastasis in a spontaneous model of breast cancer induced to develop arthritis.

## List of abbreviations

(AA): Autoimmune arthritis; (CII): Type II collagen; (CIA): Collagen-induced arthritis; (CFA): Complete Freund's adjuvant; (IL-17): Interleukin-17; (IL-6): Interleukin-6; (Pro-MMP9): Pro- Matrix metallopeptidase 9; (GF-II): Insulin like growth factor-II; (M-CSF): Macrophage colony stimulating factor; (PCNA): Proliferating cell nuclear antigen; (COX-2): Cyclooxygenase-2; (VEGF): Vascular endothelial growth factor.

## Competing interests

The authors declare that they have no competing interests.

## Authors' contributions

LDR designed and carried out the experiments, and wrote the manuscript. LP and TT helped with the dissections and endpoints. HEG interpreted the x-ray imaging. SG conducted the quantification of the IHC images. PM is the principal investigator of the laboratory in which the research was performed and contributed to the interpretation of the data and writing of the manuscript.

All of the authors have read and have approved the final manuscript.

## Authors' information

Pinku Mukherjee, PhD, Irwin Belk Distinguished Professor of Cancer Research, Department of Biology, University of North Carolina, Charlotte, NC. Dr Mukherjee has worked on Breast Cancer for the past 22 years.

Lopamudra Das Roy, PhD, Research assistant professor, Department of Biology, University of North Carolina, Charlotte, NC. Dr Roy has received funding for her work in Breast Cancer Research from The US Department of Defense.

Sriparna Ghosh, PhD, Director, Microscopy and Imaging Core Facility, Carolinas Medical Center, Charlotte, NC. Dr Ghosh has over 10 years of experience in breast, pancreatic, multiple myeloma and testicular cancer research.

Latha Pathangey, MSc, senior technologist, Mayo Clinic Arizona, Department of Biochemistry/Molecular Biology, Scottsdale, AZ. Ms Pathangey has worked with Dr Mukherjee for five years at Mayo Clinic.

Teresa Tinder, BSc, senior technologist, Mayo Clinic Arizona, Department of Immunology, Scottsdale, AZ has worked with Dr Mukherjee for 10 years.

Helen E. Gruber, PhD, Director, Biology Division, Department of Orthopedic Surgery, Carolinas Medical Center, Charlotte, NC. Dr Gruber has over 27 years of experience in the area of bone pathology and osteoarthritis and bone metastasis.

## Pre-publication history

The pre-publication history for this paper can be accessed here:

http://www.biomedcentral.com/1471-2407/11/365/prepub
